# Recording ten-fold larger I_Kr_ conductances with automated patch clamping using equimolar Cs^+^ solutions

**DOI:** 10.3389/fphys.2024.1298340

**Published:** 2024-01-24

**Authors:** Meye Bloothooft, Bente Verbruggen, Fitzwilliam Seibertz, Marcel A. G. van der Heyden, Niels Voigt, Teun P. de Boer

**Affiliations:** ^1^ Department of Medical Physiology, Division of Heart and Lungs, University Medical Center Utrecht, Utrecht, Netherlands; ^2^ Institute of Pharmacology and Toxicology, University Medical Center Göttingen, Georg-August University Göttingen, Göttingen, Germany; ^3^ DZHK (German Center for Cardiovascular Research), Partner Site Göttingen, Göttingen, Germany; ^4^ Cluster of Excellence “Multiscale Bioimaging: from Molecular Machines to Networks of Excitable Cells” (MBExC), University of Göttingen, Göttingen, Germany; ^5^ Nanion Technologies GmbH, Munich, Germany

**Keywords:** automated patch clamp, cardiac electrophysiology, Cs^+^, drugs, hERG, conductance, ion channel, hiPSC-CM

## Abstract

**Background:** The rapid delayed rectifier potassium current (I_Kr_) is important for cardiac repolarization and is most often involved in drug-induced arrhythmias. However, accurately measuring this current can be challenging in human-induced pluripotent stem cell (hiPSC)-derived cardiomyocytes because of its small current density. Interestingly, the ion channel conducting I_Kr_, hERG channel, is not only permeable to K^+^ ions but also to Cs^+^ ions when present in equimolar concentrations inside and outside of the cell.

**Methods:** In this study, I_hERG_ was measured from Chinese hamster ovary (CHO)-hERG cells and hiPSC-CM using either Cs^+^ or K^+^ as the charge carrier. Equimolar Cs^+^ has been used in the literature in manual patch-clamp experiments, and here, we apply this approach using automated patch-clamp systems. Four different (pre)clinical drugs were tested to compare their effects on Cs^+^- and K^+^-based currents.

**Results:** Using equimolar Cs^+^ solutions gave rise to approximately ten-fold larger hERG conductances. Comparison of Cs^+^- and K^+^-mediated currents upon application of dofetilide, desipramine, moxifloxacin, or LUF7244 revealed many similarities in inhibition or activation properties of the drugs studied. Using equimolar Cs^+^ solutions gave rise to approximately ten-fold larger hERG conductances. In hiPSC-CM, the Cs^+^-based conductance is larger compared to the known K^+^-based conductance, and the Cs^+^ hERG conductance can be inhibited similarly to the K^+^-based conductance.

**Conclusion:** Using equimolar Cs^+^ instead of K^+^ for I_hERG_ measurements in an automated patch-clamp system gives rise to a new method by which, for example, quick scans can be performed on effects of drugs on hERG currents. This application is specifically relevant when such experiments are performed using cells which express small I_Kr_ current densities in combination with small membrane capacitances.

## 1 Introduction

The rapid delayed rectifier potassium current (I_Kr_) largely defines cardiac repolarization (Li et al., 1996). Dysfunction of the ion channel conducting I_Kr_, the human ether-à-go-go-related gene (hERG) channel, can cause QT interval prolongation on an ECG. This prolongation may result in lethal ventricular arrhythmia, like Torsade de Pointes (Sanguinetti et al., 1995; Sanguinetti and Tristani-Firouzi, 2006). Therefore, interference by candidate drugs with the I_Kr_ function is a major reason for halting drug development (Haverkamp et al., 2000). Strict guidelines (ICH S7b) were set up for testing new drugs on their potential effect on the hERG channel (Center for Biologics Evaluation and Research and Research CfDEa, 2005). A reliable pipeline for cardiac safety testing drugs on hERG is of pivotal importance for drug safety testing (Hancox et al., 2008; Heijman et al., 2014).

Testing drugs on experimental animals has disadvantages due to physiological differences with humans, in addition to the ethical debate on animal use. Being able to test drugs on human cells or tissues would bypass animal use and give a much more reliable and relevant image of the effect on humans (Van Norman, 2019; Seibertz et al., 2023a). The comprehensive *in vitro* proarrhythmia assay (CiPA) was proposed to assess preclinical proarrhythmic liabilities of new drugs using human-based model systems. It relies on high-throughput patch clamping to characterize effects of drugs on individual ion channel types, which is used to predict drug effects with action potential computer models. In the following steps, drug effects are further investigated in human-induced pluripotent stem cell (hiPSC)-derived cardiomyocytes (CM) and ECG studies in healthy humans. This drug testing approach should result in the acceptance of drugs with few cardiac side effects and ultimately few retractions from the market (Sager et al., 2014).

However, to relate findings of drug effects on I_Kr_ in heterologous expression cell lines to hiPSC-CM is difficult, particularly on a high-throughput automated patch clamp platform. Stable human embryonic kidney (HEK)-293- or Chinese hamster ovary (CHO) cell lines overexpressing hERG1a display large current densities that can be reliably studied. In contrast, hERG1a expression levels in native cardiomyocytes and hiPSC-derived cardiomyocytes are very low (Goversen et al., 2019; Seibertz et al., 2023b). Normalized to membrane capacitance, a maximal I_Kr_ density is in the order of 1 pA/pF, which translates to absolute values of ∼150 pA and ∼30 pA in adult cardiomyocytes and hiPSC-CM, respectively. This small current makes it hard to test the effect of drugs precisely, due to technical artefacts, such as leak current, or physiological artefacts, like channel specificity (Goversen et al., 2019).

A unique property of I_Kr_ channels is that they not only conduct K^+^ ions but, under certain conditions, also Cs^+^ ions (Schönherr and Heinemann, 1996). Pipette and bath solutions containing equimolar Cs^+^ in patch-clamp experiments have been used before in HEK-hERG cells (Zhang et al., 2003; Zhang, 2006), rabbit ventricular cardiomyocytes (Zhang, 2006), neonatal rat ventricular myocytes (Dennis et al., 2012), canine ventricular myocytes (Nalos et al., 2011), and iPSC-CM (Goversen et al., 2019). The important advantage of Cs^+^ over K^+^ is that cesium blocks most other ion channels in the heart (Youm et al., 2000). This allows the recording of relatively pure hERG channel-specific currents. In addition, the previously mentioned studies that measured hERG currents with Cs^+^ solutions are estimated to be about five- to ten-fold larger than with K^+^ solutions. Cs^+^ solutions might, therefore, be more suitable to measure changes in the current due to drug block and adjust for technical artifacts. Replacing K^+^ solutions with Cs^+^ solutions in patch clamp experiments might obtain more specific data on the impact of drugs on I_Kr_.

In this study, we followed up on our earlier work using equimolar Cs^+^ solutions for a manual patch clamp in CHO cells, native cardiomyocytes, and hESC/hiPSC-CM (Nalos et al., 2011; Goversen et al., 2019) by implementing the approach on automated patch-clamping platforms. Using an automated patch-clamp system, we compared hERG currents recorded from CHO-hERG1A cells using K^+^- and Cs^+^-based solutions, and evaluated how these currents are affected by dofetilide, desipramine, moxifloxacin, and LUF-7244. Furthermore, we tested hERG currents on hiPSC-CM with Cs^+^ solutions on an automated patch-clamp system. We observed a good correlation between drug effects using both charge-carrying ions and a ten-fold larger conductance density using Cs^+^ solutions.

## 2 Materials and methods

### 2.1 Cell culture and sample preparation

hERG1A protein was stably expressed in a Chinese hamster ovary cell line (CHO cre/lox hERG, ATCC reference Nr. PTA-6812, kindly provided by Dr. Polonchuk, Hoffmann–La Roche, Basel, Switzerland). The cells were cultured in an F-12 medium (11765-054, Gibco, Waltham, United States) supplemented with 10% fetal calf serum (35-015-CV, Corning, United States), 50 U/mL penicillin, and 50 μg/mL streptomycin (30-002-CI, Corning, United States). A cell passage was performed twice a week and kept under 20 passages for optimal cell quality. Before use, the cells were washed twice with PBS (10010-015, Gibco, Waltham, United States) and dissociated with 0.3 mL Trypsin-Versene mixture (17-161E, Lonza, Basel, Switzerland) for 5 min at 37°C. Thereafter, the cells were resuspended in 1.7 mL external K^+^ solution.

The hiPSC line iUMGi014-C clone 14 (isWT1.14) was differentiated into ventricular hiPSC-CM using standard protocols (Cyganek et al., 2018). They were cultured with RPMI 1640 (72400047, Gibco, Waltham, United States) supplemented with B27 (A3582801, Gibco, Waltham, United States). Before use, the cells were washed twice with PBS (10010-015, Gibco, Waltham, United States) and dissociated with TrypLE™ enzyme (12604013, Gibco, Waltham, United States) for 10 min at 37°C. The cells were then resuspended in divalent-free HBSS (14170070, Gibco, Waltham, United States) prior to measurement. All experimental protocols using hiPSC-CM were approved by the ethics committee of the University Medical Center Göttingen (10/9/15).

### 2.2 Automated patch-clamp experiments

The composition of the different patch-clamp solutions is shown in Table 1. The solutions were based on Zhang (2006), and we added fluoride to our solutions to enhance the seal and improve the experimental outcome.

**TABLE 1:** Compositions of patch-clamp internal, external, and seal enhancer solutions.

	K^+^		Cs^+^		
Compound (mM)	Internal	External	Internal	External	Seal enhancer
NaCl	10	140	-	-	130
KCl	10	4	-	-	4
KF	110	-	-	-	-
CaCl_2_	-	2	-	10	10
MgCl_2_	-	1	1	1	1
D glucose-monohydrate	-	5	-	10	5
HEPES	10	10	10	10	10
EGTA	10	-	10	-	-
CsCl	-	-	10	135	-
CsF	-	-	125	-	-
pH	7.2	7.4	7.2	7.4	7.4
Adjusted with	KOH	NaOH	CsOH	NaOH	NaOH

After sample preparation, experiments on CHO cells were performed at 22°C on the Nanion^®^ Patchliner system (Nanion Technologies, Munich, Germany) with a EPC-10 Quadro Amplifier (HEKA, Reutlingen, Germany) controlled by Patchmaster 2.90.5. Recordings were performed using membrane capacitance and series resistance compensation (70%). The chip (NPC-16 medium resistance, Nanion Technologies) was filled with either the K^+^ or Cs^+^ internal and external solution with a resistance between 2 and 4 MOhm. Thereafter, the cells were added to the chip. After the capture of a cell, the seal enhancer solution was added, obtaining a R_seal_ of at least 200 MOhm. Next, the chip was flushed with an external solution. During the experiment, four cells were measured simultaneously in separate chambers of the chip.

Experiments on hiPSC-CM were performed on a Nanion^®^ SyncroPatch 384 system (Nanion Technologies, Munich, Germany) with a thin borosilicate glass and single-aperture 384-well chips (NPC384T 1 x S-type, Nanion Technologies, Munich, Germany). A single run on a partial plate was performed at 22°C using Cs^+^ internal and external solutions. A seal enhancer was applied before final external solution addition. Currents were recorded with an integrated amplifier controlled by PatchControl 384 software (Nanion Technologies, Munich, Germany).

For all experiments, the drugs were incubated for 60 s before measuring and an activation pulse of +20 mV and −80 mV both for 500 m was given to open the channel and achieve the steady-state ion channel–drug interaction.

The K^+^ and Cs^+^ solutions give rise to very different I_Kr_ reversal potentials (−86.5 and 0.0 mV, respectively); therefore, the voltage clamp protocols were adapted to fit both situations, as in previously validated work by Zhang (2006). For Cs^+^-based experiments, the cells were depolarized from a holding potential of −80 mV in 10-mV increments between −70 mV and +40 mV for 3 s. Thereafter, the cell was clamped back to −80 mV for 2 s, followed by a hyperpolarization step to −100 mV for 50 ms before returning to −80 mV. In K^+^-based experiments, the cells were clamped at a holding potential of −80 mV before a hyperpolarizing step to −120 mV for 10 ms and returned to −80 mV for 50 ms before cells were depolarized from −60 mV to 60 mV in 10-mV increments for 1 s. Thereafter, the cells were clamped back to −50 mV for 500 ms and finally returned to −80 mV. The interval between sweeps was 10 s for both protocols.

### 2.3 Compounds

Different concentrations of dofetilide (0.3–10 µM), desipramine (1–1,000 µM), moxifloxacin (10–300 µM), and LUF7244 (1–30 µM) were tested in CHO cells and E4031 (10 µM) in hiPSC-CMs. For the stock solutions, the drugs were dissolved in ddH_2_O and for the working solution diluted in either the K^+^ or Cs^+^ external solution. The different concentrations per compound were sequentially tested on the same cell.

### 2.4 Data analysis

For all measured currents, the current densities (pA/pF) were determined by normalizing for the membrane capacitance. The current densities were converted to conductance densities (pS/pF) using reversal potentials calculated using the Nernst equation for the solution and temperature used.

Dose–response curves were made with the tail current values at 40 mV, which were the largest current densities. The curves were fitted with non-linear regression with the Hill equation, and absolute IC_50_ values were estimated. hiPSC hERG currents were analyzed offline by DataControl 384 software (Nanion Technologies, Munich, Germany) (Seibertz et al., 2022).

### 2.5 Statistics

All averages are expressed as mean ± SEM for the (maximal) conductance values and IC_50_ values. To determine the amount of block, a two-way ANOVA with Dunnett’s test was performed with the maximal conductance values. To compare the difference of the hERG conductance for the two different solutions, a Mann–Whitney test was performed on all the unblocked data for the maximal conductance.

## 3 Results

In this study, the effect of the use of Cs^+^ solutions to measure hERG conductance was compared to the regularly used K^+^ solutions. This was conducted in an ectopic cell system (CHO-hERG) and a human cell system (hiPSC-CM). Since hERG is a common drug target, the effect of different compounds was tested. To obtain high-throughput data, patch-clamp experiments were performed on automated systems.

### 3.1 Desipramine

**FIGURE 1:**
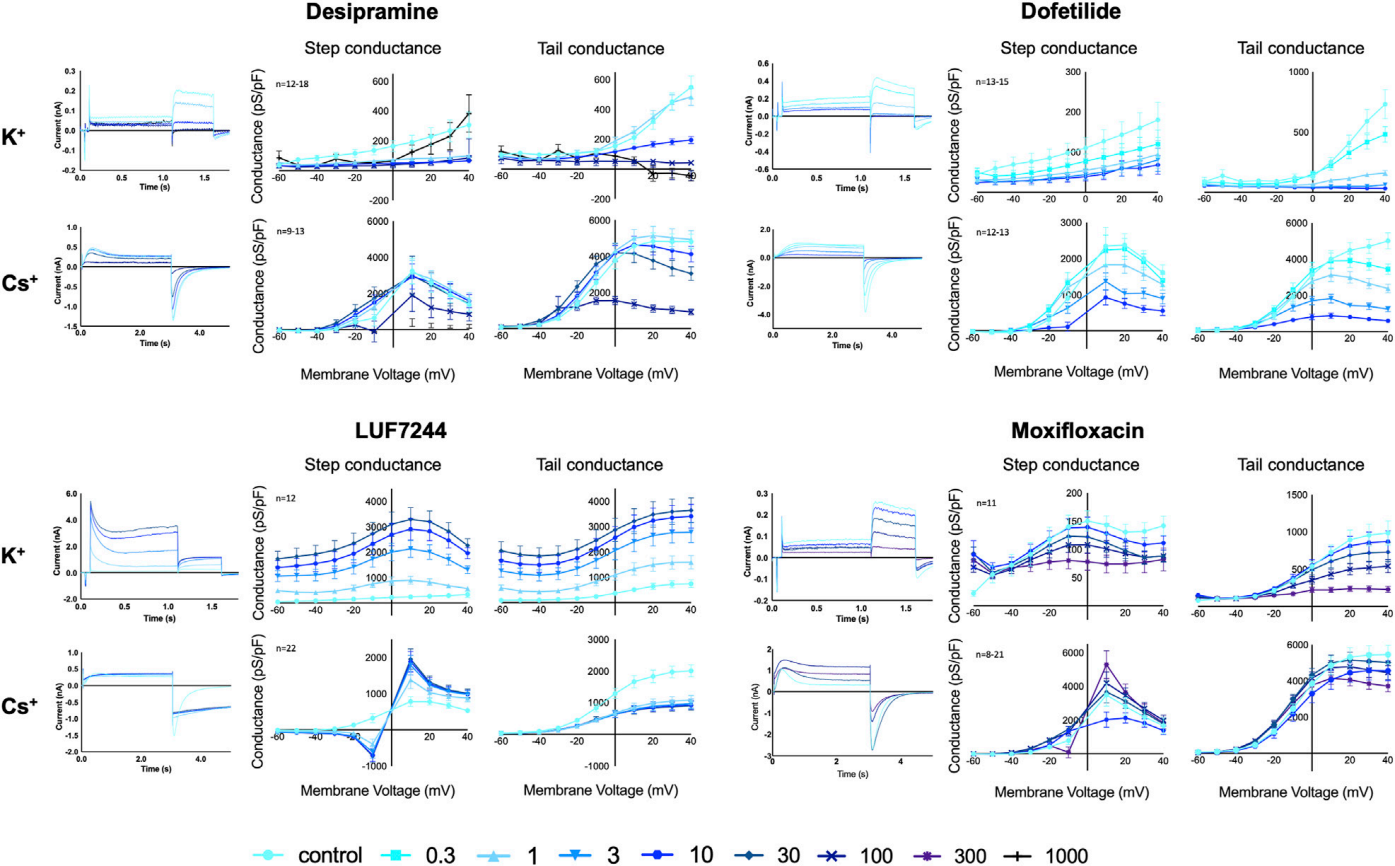
hERG Current traces and conductance for K^+^ and Cs^+^ solutions with different concentrations of desipramine, dofetilide, LUF7244, and moxifloxacin treatment in CHO-hERG cells. Data are shown as mean ± SEM.

Desipramine is a tricyclic drug used for the treatment of depression and is known to acutely block hERG (Staudacher et al., 2011). The hERG conductance for both the K^+^ and the Cs^+^ solutions decreased upon desipramine treatment in a concentration-dependent manner; see Figure 1. The conductance of the step current, K^+^ step conductance, was almost completely inhibited at 1 µM (*p* = 0.0028). However, with the 1,000 µM treatment, the conductance was similar as the control measurement again, although the standard deviation for this concentration was quite large. The conductance of the tail current, K^+^ tail conductance, was completely blocked with 100 and 1,000 µM desipramine (both *p*=<0.0001). For the Cs^+^ solutions, upon the highest drug incubation (100 µM), the step conductance was slightly decreased (*p* = 0.049), and the tail conductance showed a strong decrease but not a complete block (*p*=<0.0001).

### 3.2 Dofetilide

Dofetilide is a well-known class III antiarrhythmic drug and blocks hERG (Ficker et al., 1998). Upon dofetilide treatment, the conductance with both K^+^ and Cs^+^ solutions decreased with increasing compound concentrations; see Figure 1. With the K^+^ solutions, the step conductance decreased upon dofetilide treatment, and from 3 µM onward, a significant block was seen (*p* = 0.033), without a complete block. The K^+^ tail conductance also diminished in a dose-dependent manner. From 0.3 µM, there was a significant block (*p*=<0.0001), with a complete block for concentrations above 3 µM. With the Cs^+^ solutions, the step and tail conductance decreased significantly from, respectively, 3 μM and 0.3 µM dofetilide incubation (*p* = 0.0002 and *p*=<0.0001) in a concentration-dependent manner, but at 10 µM, no complete block was established.

### 3.3 LUF7244

LUF7244 is an allosteric modulator of hERG, which, upon treatment, results in very large hERG currents (Qile et al., 2019). In our experiments, LUF7244 was an activator of the hERG conductance for both K^+^ and Cs^+^ solutions, but the activation was dose-dependent with K^+^ solutions and dose-independent with Cs^+^ solutions; see Figure 1. Using K^+^ solutions, there was a strong dose-dependent increase for both step and tail conductance, which was significant from 3 µM onward (*p*=<0.0001 for both). For the K^+^ step conductance, the maximal conductance increased ×10.1 between control and 30 μM LUF7244; for the tail conductance, the increase was 4.9x. This large increase was already well-known for this compound together with a change in the current trace morphology (Qile et al., 2019). For the Cs^+^ solutions, the step conductance was lower compared to control at voltages below the reversal potential (0 mV) and higher when tested above the reversal potential (1 µM *p* = 0.0007) but not in a dose-dependent manner. These changes were relatively small compared to the large increase when using the K^+^ solutions. Interestingly, the peak Cs^+^ tail conductance decreased upon treatment (1 µM *p*=<0.0001) but not dose-dependent, which is opposite to the experiments using K^+^ solutions. In addition, a change in the current trace morphology is seen. The current trace shows the mentioned lower peak tail current but a higher area under the curve when comparing the control measurement to LUF7244 measurements. This morphology change is likely a result of altered channel kinetics since both Cs^+^ and LUF7244 might alter hERG channel inactivation, and combining the results of LUF7244 with Cs^+^ and K^+^ solutions shows that Cs^+^ solutions are not always the perfect fit for drug interaction testing on I_hERG_.

### 3.4 Moxifloxacin

The antibiotic moxifloxacin is associated with modest QT prolongations due to the hERG channel block (Alexandrou et al., 2006). The step and tail conductance with the K^+^ solutions decreased upon treatment (see Figure 1) although the inhibition for the tail conductance was stronger (100 μM, step: *p* = 0.0580, tail: *p* = 0.0003).

With the Cs^+^ solutions, the step conductance decreases for compound incubations until 10 μM but increased a little compared to control upon 100 µM and significantly at 300 µM incubation (*p* = 0.0003). The Cs^+^ tail conductance showed a slight decrease in the conductance upon treatment (300 µM *p*=<0.0001). However, in general, the effect of the drug was smaller with Cs^+^ solutions compared to the K^+^ solutions.

### 3.5 Maximal hERG current conductance is higher with equimolar Cs^+^ solutions

As depicted in Figure 1, in absence of I_hERG_ blocking or enhancing drugs, the conductance of the Cs^+^ carried current was larger than the conductance of the K^+^-carried current. A comparison of the combined unblocked maximal hERG conductance data (*n* = 56 for K^+^ and *n* = 69 for Cs^+^) showed that the Cs^+^-carried step conductance was 10.2-fold larger (*p* < 0.0001, K^+^ mean = 296 ± 40 pS/pF, Cs^+^ mean = 2366 ± 284 pS/pF) and the Cs^+^-carried tail conductance was 5.7-fold larger (*p* < 0.0001, K^+^ mean = 726 ± 62 pS/pF, Cs^+^ mean = 4152 ± 272 pS/pF), and all statistical data are available in Supplementary Table S1.

### 3.6 Dose–response curves

**FIGURE 2:**
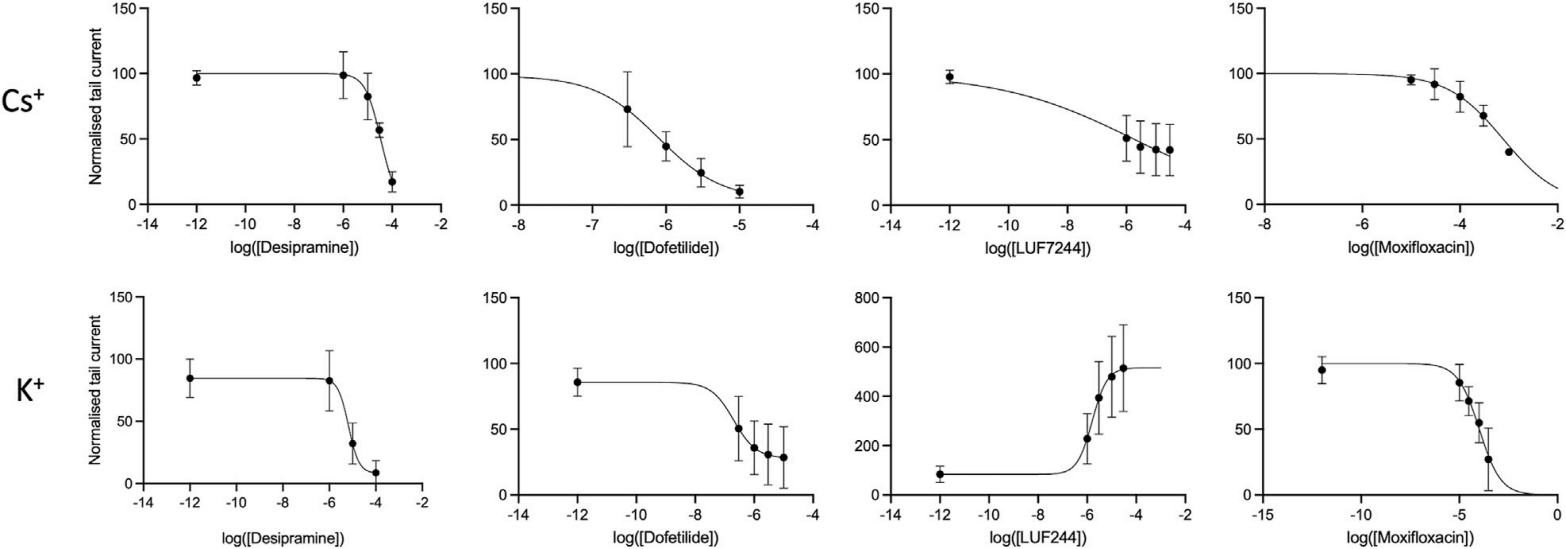
Concentration response curves for different drugs with either Cs^+^ or K^+^ solutions of the fully activated hERG current at + 40 mV.

Dose–response curves of the tail current amplitude with the drugs at tested concentrations were made and fitted with a Hill equation to obtain IC_50_ values; see Figure 2. In addition, the log (IC_50_) and Hill coefficients were estimated, as shown in Table 2.

**TABLE 2:** IC_50_ values and Hill coefficients of the different drugs for either Cs^+^ or K^+^ solutions.

	K^+^			Cs^+^		
	Log(IC_50_)	Conc. (µM)	Hill coef.	Log(IC_50_)	Conc. (µM)	Hill coef.
Desipramine	−5.2	6.9	−1.4	−4.4	33.9	−1.4
Dofetilide	−6.7	0.2	−1.1	−6.1	0.8	−1.0
LUF7244	−5.8 (EC_50_)	1.6	1.4	−5.7	2.9	−0.2
Moxifloxacin	−4.0	103.4	−0.8	−3.1	759.7	−0.8

As is already clear from Figure 1, dofetilide is the strongest hERG current inhibitor, followed by desipramine and moxifloxacin, which show a modest inhibition. As is already apparent from the maximal conductance analysis between Cs^+^ and K^+^ solutions, the IC_50_ values show that the inhibition with K^+^ solutions is stronger than that with the Cs^+^ solutions. This indicates that the effect of measuring drug inhibition of I_hERG_ using Cs^+^ solutions underestimates the drug effect.

For LUF7244, the morphology of the current traces changes upon treatment. With the Cs^+^ solutions, the maximal tail current declines upon treatment, and however, the total area under the curve is larger compared to control. Given that IC_50_ is calculated for the maximal tail current, this gives a skewed image of the effect of the drug. Considering the relative log (IC_50_) value, the value is −6.5 (compared to −5.7 for the max current) with a corresponding concentration of 0.3 µM.

### 3.7 Cs^+^-based hERG current in hiPSC-derived cardiomyocytes

**FIGURE 3:**
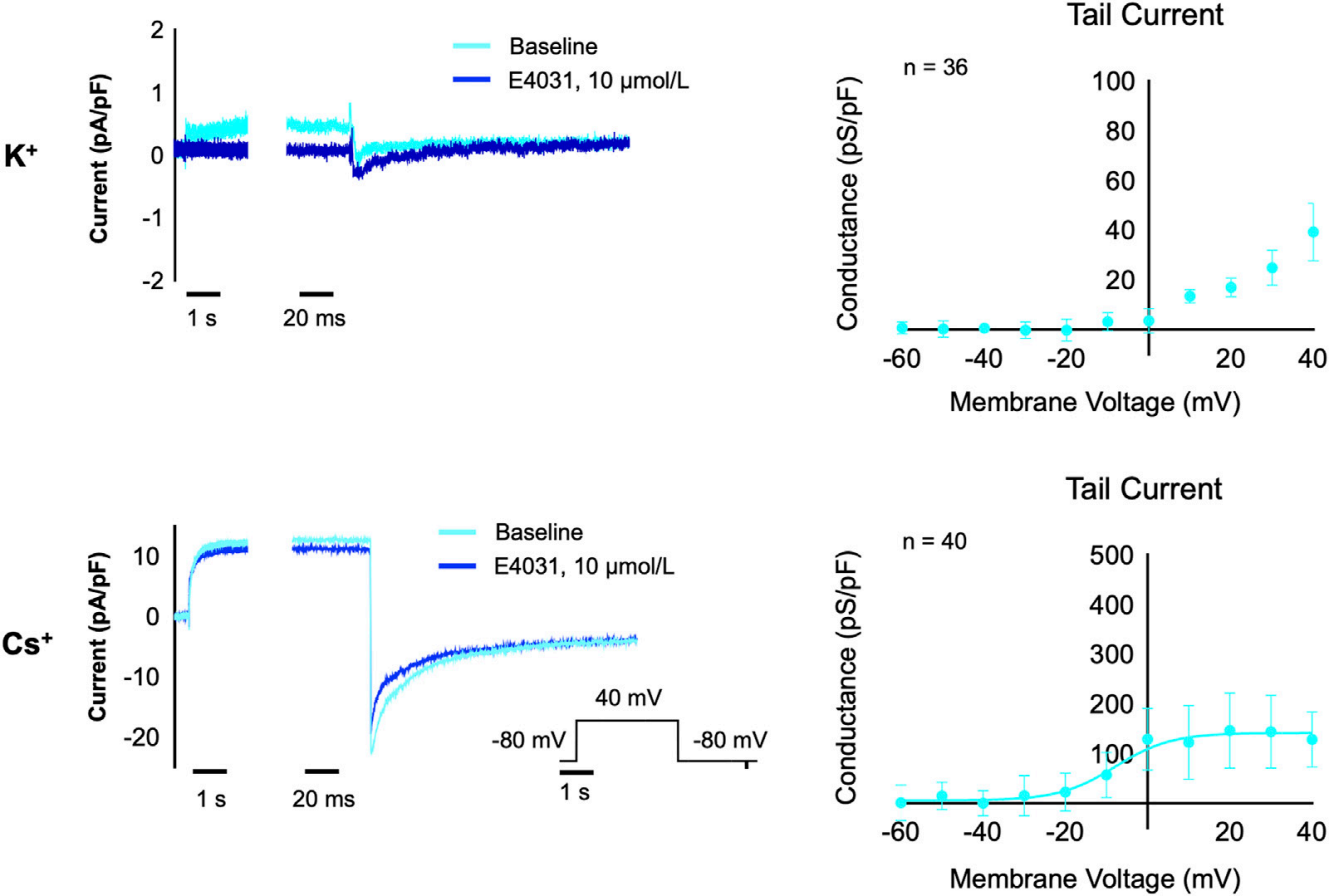
I_Kr_ measurements in hiPSC-CM with K^+^ solutions (*n* = 36) and Cs^+^ solutions (*n* = 40). The left panels show a current trace for baseline (light blue) and after acute treatment with the hERG inhibitor E4031 (10 µM, dark blue) in K^+^ solutions (upper) and Cs^+^ solutions (lower). The right panels show the relation between test potential and conductance of the E4031-sensitive component of the tail current in K^+^ solutions (upper) and Cs^+^ solutions (lower).

Next, we aimed to test if the equimolar Cs^+^ solution can also be used to record I_Kr_ in hiPSC-CM on an automated patch-clamp platform. With these solutions, we observed a relatively large I_Kr_, which could be inhibited by the hERG inhibitor E4031, as shown in Figure 3. The conductance of the measured tail current in hiPSC-CM is approximately half of the K^+^ conductance observed in the CHO-hERG cells and approximately one tenth of the Cs^+^ conductance in CHO-hERG cells, reflecting the high expression levels of hERG ion channels in the CHO-hERG cell line.

## 4 Discussion

The hERG channel underlies I_kr_, which allows potassium ion movement out of cells and thereby induces the repolarization of the heart. Because many (candidate) drugs interact with the hERG channel and therefore hamper repolarization, testing interference with the hERG function is an important component of the assessment of drug safety. Generally, the hERG current is recorded in patch-clamp experiments using potassium as the charge carrier; however, the channel is also permeable to cesium, which gives rise to Cs^+^-based hERG currents. In this study, we measured the conductance of hERG currents in CHO-hERG cells and hiPSC-CM for the first time on automated patch-clamp systems with both regular K^+^ and equimolar Cs^+^ solutions. The current traces measured here are very comparable to Cs^+^-based currents measured previously (Zhang et al., 2003; Zhang, 2006). We show that the hERG channel conductance with equimolar Cs^+^ solutions is on average 10 times higher compared to the conductance with K^+^ solutions.

Upon drug incubation, the conductance between K^+^ and Cs^+^ is affected in a similar pattern, *e.g.*, inhibition, although the effect with K^+^ solutions on average is larger. This is in line with a previous work that showed a strong hERG block upon drug incubation of known hERG blockers when using Cs^+^ solutions (Zhang, 2006).

However, with an activator compound, LUF7244, the results are less comparable. The experiments with K^+^ solutions show the known large increase in conductance, and however, experiments with Cs^+^ solutions show the opposite effect: an inhibition of hERG conductance. This difference can be explained by a change in tail current morphology due to altered channel gating. As shown in our previous work (Qile et al., 2019), this might be the result of a change in inactivation; however, the mechanism of LUF7244 action is not fully understood. Given that presence of Cs^+^ ions alters inactivation kinetics (Zhang et al., 2003), the use of Cs^+^ solutions to test drugs that impact inactivation (such as LUF7244) should be performed with caution.

Desipramine, dofetilide, and moxifloxacin are inhibitors of I_hERG_. These drugs show a concentration-dependent decrease in conductance for the two different types of solutions. Interestingly, the relative amount of inhibition is somewhat smaller for the Cs^+^ solutions despite the larger current magnitudes. Zhang et al. (2003) showed that the activation of the hERG channel is similar using either K^+^ or equimolar Cs^+^ solutions, but the inactivation and recovery from inactivation is slower when using equimolar Cs^+^. Slower inactivation may result in reduced acute inhibition due to state-dependent blocking effects of drugs, providing a potential explanation of the observed differences in relative inhibition strength. Further insights in these differences may be gained through a better understanding of the Cs^+^ current kinetics, for example, by creating a mathematical model of the Cs^+^ current based on individual cell recordings (Beattie et al., 2018; Lei et al., 2019).

The hERG measurements in hiPSC-CM with Cs^+^ solutions show a similar current trace as a regular K^+^-based hERG current trace. Conductance was much smaller than in CHO-hERG cells, reflecting the lower endogenous expression levels of hERG in hiPSC-CM. However, our measurements with the Cs^+^ solution demonstrate much larger tail currents compared to our experiments using K+ based solutions, similar to previous reports using K+ as a charge carrier (Ma et al., 2011; Sala et al., 2016). This demonstrates that utilizing Cs^+^ solutions increases the amount of measured current in hiPSC-CM. Following a full block with E4031, residual tail current is still present in hiPSC-CM recordings. As hiPSC-CM contains a variety of ionic channels, it is possible that this artefact is also influenced by chloride currents, such as I_Cl(Ca)_, which can hinder reliable I_Kr_ measurements in hiPSC-CM in the absence of specific blockers (Knollmann, 2013).

In this study, we used automated patch-clamping platforms to record the hERG current with equimolar Cs^+^ solutions and compared it with the hERG current recorded using K^+^-based solutions. The Cs^+^ currents we observed are very similar qualitatively and quantitatively to those we recorded earlier in the manual patch-clamp experiment using CHO cells, hiPSC-CM (Goversen et al., 2019), and hES-CM (Jonsson et al., 2012). A clear benefit of using equimolar Cs^+^ solutions is a much larger tail current, which is hERG-specific. Testing drug effects on hERG current yields similar results, although effects with equimolar Cs^+^ seem somewhat smaller. However, when drugs impact channel inactivation kinetics, Cs^+^ should be used with caution. Overall, using Cs^+^ as the charge carrier may prove useful in both manual and automated patch-clamp experiments when testing cells which have a relatively small hERG current, such as hiPSC-CM or native cardiac cells.

## Data Availability

The raw data supporting the conclusions of this article will be made available by the authors, without undue reservation.
