# Supplementary material for: Recording ten-fold larger IKr conductances with automated patch clamping using equimolar Cs+ solutions
Source: Front Physiol. 2024 Jan 24;15:1298340. doi: 10.3389/fphys.2024.1298340 (PMC10847579; doi:10.3389/fphys.2024.1298340)
Supplement: Supplementary file 1 [file DataSheet1.PDF]

Supplemental 1. Statistical analysis of CHO-hERG conductance data

Potassium step conductance

Desipramine (40 mV)	
control vs. 1	0.0028
control vs. 10	0.0004
control vs. 100	0.0014
control vs. 1000	0.6911
Dofetilide (40 mV)	
control vs. 0.3	0.3124
control vs. 1	0.0744
control vs. 3	0.0326
control vs. 10	0.0124
LUF7244 (10 mV)	
control vs. 1	0.2002
control vs. 3	<0,0001
control vs. 10	<0,0001
control vs. 30	<0,0001
Moxifloxacin (40 mV)	
control vs. 10	0.4525
control vs. 30	0.059
control vs. 100	0.058
control vs. 300	0.0274

Cesium step conductance

Desipramine (10 mV)	
control vs. 1	>0,9999
control vs. 10	0.9732
control vs. 30	0.9928
control vs. 100	0.0494
Dofetilide (10 mV)	
control vs. 0.3	0.9737
control vs. 1	0.099
control vs. 3	0.0002
control vs. 10	<0,0001
LUF7244 (10 mV)	
control vs. 1	0.0007
control vs. 3	<0,0001
control vs. 10	<0,0001
control vs. 30	<0,0001
Moxifloxacin (10 mV)	
control vs. 10	0.0611
control vs. 30	0.995
control vs. 100	0.3884
control vs. 300	0.0003

Potassium tail conductance

Desipramine (40 mV)	
control vs. 1	0.5144
control vs. 10	<0,0001
control vs. 100	<0,0001
control vs. 1000	<0,0001
Dofetilide (40 mV)	
control vs. 0.3	<0,0001
control vs. 1	<0,0001
control vs. 3	<0,0001
control vs. 10	<0,0001
LUF7244 (40 mV)	
control vs. 1	0.1957
control vs. 3	<0,0001
control vs. 10	<0,0001
control vs. 30	<0,0001
Moxifloxacin (40 mV)	
control vs. 10	0.6896
control vs. 30	0.0743
control vs. 100	0.0003
control vs. 300	<0,0001

Cesium tail conductance

Desipramine (40 mV)	
control vs. 1	0.9989
control vs. 10	0.3648
control vs. 30	0.0008
control vs. 100	<0,0001
Dofetilide (40 mV)	
control vs. 0.3	<0,0001
control vs. 1	<0,0001
control vs. 3	<0,0001
control vs. 10	<0,0001
LUF7244 (40 mV)	
control vs. 1	<0,0001
control vs. 3	<0,0001
control vs. 10	<0,0001
control vs. 30	<0,0001
Moxifloxacin (40 mV)	
control vs. 10	0.3351
control vs. 30	0.7205
control vs. 100	0.0513
control vs. 300	<0,0001

Statistical analysis outcome of the2-way ANOVA comparing the different concentrations of different compounds with the control measurement. The comparison has been executed for the highest conductance (membrane potential between brackets behind the compound name). Values highlighted in yellow represent the concentration significantly different from control ($p<0.05$).
